# Supplementary material for: Expected features of the course leader in the rehabilitation healthcare professionals’ higher education: A qualitative study on students’ perspectives
Source: PLoS One. 2024 Dec 17;19(12):e0312943. doi: 10.1371/journal.pone.0312943 (PMC11651607; doi:10.1371/journal.pone.0312943)
Supplement: Attachment — Submitted filename: Manuscript PLOS One Manuscript PONE-D-24-09846 feedback.docx [file pone.0312943.s001.docx]

PLOS ONE Peer-review

Article title: **Expected Features of the Course Leader in the Rehabilitation Healthcare Professionals’ Higher Education: A Qualitative Study on Students’ Perspectives**

Manuscript ID: **PONE-D-24-09846**

Thank you for providing me the opportunity to review this manuscript. This article is a much-needed reflection on the multi-faceted and often underappreciated role of Course Leaders teaching in rehabilitation science. I feel this manuscript could make a meaningful contribution to this under-recognized, but important conversation. I have included some feedback below for your consideration.

1. Lines 52-53:
   - Recommend editing this sentence for enhanced clarity of meaning. *“The environment in which the CLs operate within the rehabilitation healthcare professionals’ 53 higher education (encompassing undergraduate and postgraduate degree courses for healthcare 54 professionals in rehabilitation) is challenging.”*
2. Line 72:
   - Delete ‘starting’ as this is the only perspective explored in this article, correct?
3. Line 91:
   - Provide definition (length of time) for ‘recent’ graduate or include in demographic information in results section.
4. Lines 112- 114:
   - At the beginning of this paragraph you only mention three researchers: SB, LF, student. Then in these lines you state:
     “*This folder was accessible from all researchers but LF and SB, so they would not know the names of the students who decided to take part in the study and the content of their interviews until they had been transcribed and anonymised.*”
     Are there other researchers or should this state: This folder was accessible only to the student researcher, to provide anonymity from LF and SB who are members of faculty.
5. Line 125
   - Participants’ experiences
6. Line 129:
   - This is the first mention of ‘BG’. Please provider earlier – I assume this is the initials of the student? Also remove ‘a’ before ‘BG’.
7. Lines: 125- 129 and 132- 137:
   - I feel the methodology could be more clearly articulated in these lines.
   - I commend your commitment to reflexivity and keeping a reliable audit trail.
   - Table 2 is also a clear and helpful way to document your qualitative methods and the contribution of each author.
8. Line 234:
   - an MSc – edit to a MSc
9. Line 235
   - Experiences
10. Line 244:
    - priories - priorities?
11. Line 267:
    - Students’ perspectives
12. Line 286- 287:
    - *“However, it must be noted that the academic and clinical roles should not 287 overcome the CL role [20].”*
      It is not clear to me what this statement means. How would these roles ‘overcome the CL role”? Please revise and add a little more explanation for clarity.
13. Discussion:
    - You have done a nice job of summarizing and discussing your findings in the context of the literature. I would be very interested to learn what application you feel this information will provide to those in higher education (faculty and leadership) and where to from here. Perhaps another paragraph or two to flesh this out would be very meaningful to the progression of the conversation around this research topic.
14. Line 313- 314
    - In live 109, it is stated only one researcher gathered information. Were those acknowledged also involved in data gathering?
